# Oxidative Stress in Preterm Infants: Overview of Current Evidence and Future Prospects

**DOI:** 10.3390/ph13070145

**Published:** 2020-07-07

**Authors:** Raffaele Falsaperla, Filadelfo Lombardo, Federica Filosco, Catia Romano, Marco Andrea Nicola Saporito, Federica Puglisi, Ettore Piro, Martino Ruggieri, Piero Pavone

**Affiliations:** 1 Neonatal Intensive Care, AUO San Marco-Policlinico, University of Catania, 95123 Catania, Italy; raffaelefalsaperla@hotmail.com (R.F.); marcosaporito@hotmail.com (M.A.N.S.); federicapuglisi@gmail.com (F.P.); 2 Postgraduate Training Program in Pediatrics, Department of Clinical and Experimental Medicine, University of Catania, Catania street Santa Sofia 78, 95123 Catania, Italy; delfo.lombardo@gmail.com (F.L.); federica.filosco@gmail.com (F.F.); 3 Child and Adolescent Neuropsychiatry, Department Clinical and Experimental Medicine, University of Catania, 95123 Catania, Italy; catiaromano@alice.it; 4 University Hospital “P. Giaccone”, Department of Sciences for Health Promotion, Maternal Infant Care, Internal Medicine and Medical Specialties “G. D’Alessandro”, Neonatal Intensive Care Unit, 90121 Palermo, Italy; ettore.piro@unipa.it; 5 Department of Clinical and Experimental Medicine Section of Pediatrics and Child Neuropsychiatry, AUO San Marco-Policlinco, University of Catania, 95123 Catania, Italy; mruggie@unict.it

**Keywords:** oxidative stress, preterm, future prospects, review, antioxidant

## Abstract

Preterm birth (PTB), defined as parturition prior to 37 weeks of gestation, is the leading cause of morbidity and mortality in the neonatal population. The incidence and severity of complications of prematurity increase with decreasing gestational age and birthweight. The aim of this review study is to select the most current evidence on the role of oxidative stress in the onset of preterm complication prevention strategies and treatment options with pre-clinical and clinical trials. We also provide a literature review of primary and secondary studies on the role of oxidative stress in preterm infants and its eventual treatment in prematurity diseases. We conducted a systematic literature search of the Medline (Pubmed), Scholar, and ClinicalTrials.gov databases, retroactively, over a 7-year period. From an initial 777 articles identified, 25 articles were identified that met the inclusion and exclusion criteria. Of these, there were 11 literature reviews: one prospective cohort study, one experimental study, three case-control studies, three pre-clinical trials, and six clinical trials. Several biomarkers were identified as particularly promising, such as the products of the peroxidation of polyunsaturated fatty acids, those of the oxidation of phenylalanine, and the hydroxyl radicals that can attack the DNA chain. Among the most promising drugs, there are those for the prevention of neurological damage, such as melatonin, retinoid lactoferrin, and vitamin E. The microbiome also has an important role in oxidative stress. In conclusion, the most recent studies show that a strong relationship between oxidative stress and prematurity exists and that, unfortunately, there is still little therapeutic evidence reported in the literature.

## 1. Introduction

Preterm birth (PTB), defined as parturition prior to 37 weeks of gestation, is the leading cause of morbidity and mortality in the neonatal population. The incidence and severity of complications of prematurity increase with decreasing gestational age and birthweight. Every year, about 15 million babies are premature (more than one in ten of all babies born around the world). All newborns are vulnerable, but preterm babies are acutely so. Many preterm babies require special care just to remain alive. Newborn deaths—those in the first month of life—account for 40% of all deaths among children less than 5 years old. Prematurity is the world’s single biggest cause of newborn death, and the second leading cause of all child deaths, after pneumonia. Many of the preterm babies who survive face a lifetime of disability [1]. Multiple prospective studies have reported that premature newborns have higher risks of long-term neurodevelopmental disabilities, such as intellectual disability (ID), blindness, neurosensorial hearing loss, and cerebral palsy (CP). There are several risk factors accounting for prematurity, such as assisted reproductive technologies that increase the rate of multiple births (twins, triplets, or quadruples) and advanced maternal age [2]. Teenage pregnancy could also be a risk factor for prematurity, but the putative role of this factor is controversial and seems to have declined in the last 10 years [2,3]. Other maternal causes potentially related to premature births are inflammatory, hormonal, and neurochemical pathologies that could influence a mother’s ability to take appropriate care of the baby once born [4].

Some complications (e.g., necrotizing enterocolitis (NEC), retinopathy of prematurity (ROP), bronchopulmonary dysplasia (BPD), intraventricular hemorrhage (IVH)) are uncommon in late preterm infants. Most complications are related to the dysfunction of immature organ systems. In some cases, complications resolve entirely; in others, residual organ dysfunction remains [5,6]. The most common complications in extremely preterm infants are cardiac (patent ductus arteriosus (PDA)), neurological (poor sucking and swallowing reflexes, apneic episodes, IVH, developmental or cognitive delays), ocular (ROP, myopia and/or strabismus), gastrointestinal (feeding intolerance, with increased risk of aspiration, NEC), renal (metabolic acidosis, growth failure), infective (sepsis, meningitis), respiratory (respiratory distress syndrome, respiratory insufficiency of prematurity, chronic lung disease and BPD), and metabolic (hypoglycemia, hyperbilirubinemia, and metabolic bone disease—osteopenia of prematurity) [7,8,9]. These complications might have a single common denominator: oxidative stress [10]. Oxidative stress is a pathophysiological mechanism associated with spontaneous PTB, which reflects an imbalance between the systemic manifestation of reactive oxygen species (ROS) and a biological system’s ability to readily detoxify the reactive intermediates or to repair the resulting damage. 

An increase in oxidative stress has been demonstrated in infants requiring an oxygen resuscitation supplement in the delivery room [11].

Sies and Jones affirm how the increased formation of the different ROS leads to molecular damage, denoted as “oxidative distress”. The signaling and damaging properties of ROS, in particular the most prevalent and best-studied cellular oxidant H_2_O_2_ and the superoxide anion radical (O_2_^−^), represent the basis for the concept of redox homeostasis, with its components of oxidative eustress and oxidative distress. Redox signaling is universally integrated with the central homeostatic mechanisms at the molecular, organellar, cellular, tissue, and organismic levels [12].

Disturbances in the normal redox state of cells can cause toxic effects through the production of peroxides and free radicals that damage all components of the cell, including proteins, lipids, and DNA. Oxidative stress from oxidative metabolism causes base damage and DNA strand breaks. Base damage is mostly indirect and caused by ROS-generated O_2_^−^ (superoxide radical), OH (hydroxyl radical), and H_2_O_2_ (hydrogen peroxide). Indeed, the highly reactive hydroxyl radical reacts with the heterocyclic DNA bases and the sugar moiety near. At diffusion-controlled rates, hydrated electron and H atom also add to the heterocyclic bases. These reactions lead to adduct radicals, further reactions of which yield numerous products [13]. Furthermore, some ROS act as cellular messengers in redox signaling. Thus, oxidative stress can disrupt normal mechanisms of cellular signaling, and thereby cause organ damage.

Currently, an antioxidant is defined as any substance able to eliminate ROS and their derivatives [14]. In chemistry, “anti-oxidant” is simply conceived as “a compound that removes reactive species, mainly those oxygen-derived” [15]. Their functions can be classified into distinct defense lines, according to their mechanisms of action: (a) preventative agents that suppress new radicals formation (which includes enzymes, such as superoxide dismutase (SOD), catalase (CAT) and glutathione peroxidase (GPX), proteins that bind metals, like ferritin and ceruloplasmin, and minerals such as selenium (Se), copper (Cu), and zinc (Zn) [16,17]); (b) radical scavenging agents that inhibit chain initiation and/or propagation, which includes glutathione, albumin, vitamins C and E, carotenoids, and flavonoids; (c) repair and de novo enzymes that repair and reconstitute cell membranes, which include lipases, proteases, DNA repair enzymes, transferases, and methionine–sulfoxide reductases; and (d) adaptation agents that generate appropriate antioxidant enzymes and transfer them to the essential site of action [14,18]. Another category of antioxidants is peroxiredoxins (Prxs), a ubiquitous family of cysteine-dependent peroxidase enzymes that play dominant roles in regulating peroxide levels within cells. In addition, Prxs might act as modulators of inflammation, protecting against cell death and facilitating tissue repair after damage [19,20]. In addition, the potential for intracellular free radical production is greatly reduced by the ability of mitochondrial cytochrome oxidase to function catalytically in the electron transport chain without releasing ROS [8,9,10,11,12,13,14,15,16,17,18,19,20,21].

Thus, a possible correlation between oxidative stress and the development of complications in premature babies led us to perform an analysis of the available literature. Here, we synthesize recent findings on the role of oxidative stress in the onset of preterm complications, prevention strategies, and treatment options.

## 2. Search Strategy

We aimed to select the most current evidence on the role of oxidative stress in the onset of preterm complications, prevention strategies, and treatment options. We reviewed the literature (primary and secondary studies) on the role of oxidative stress in preterm infants, as well as its prevention. We searched the Medline (Pubmed), Scholar, and ClinicalTrials.gov databases retroactively over a 5-year search period. The following search terms were used: oxidative stress, free radical, antioxidant, redox, and ROS paired with preterm, preterm birth, preterm delivery, prematurity, premature birth, and premature delivery, “free radicals preterm”, “anti-oxidant preterm”, “drugs preterm”, “neurodevelopment preterm”, “pre-clinical trials preterm born”, “clinical trials preterm born”, “retinopathy of prematurity”, “necrotizing enterocolitis preterm”, and “bronchopulmonary dysplasia preterm”. The inclusion criteria were as follows: original research, written in English, and published after 2013. The exclusion criteria were as follows: pregnancy failure, abortions, stillbirths, preimplantation embryos, PTB from specific infections (e.g., malaria), maternal therapy, sepsis, the population of the clinical trial under study with less than 50 preterms, and pre-clinical trials where clinical trials do not yet exist. Twenty-five articles were selected based on the search criteria. For the sake of a rational flow of thoughts, we decided to first analyze the pre-clinical studies, then the clinical trials.

## 3. Results

**Figure 1:**
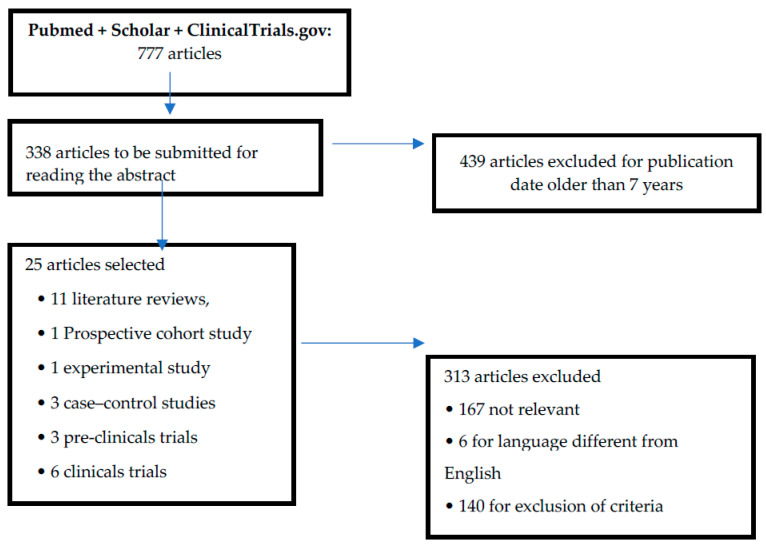
Flow diagram of the literature selection and review process.

From the 777 initially shortlisted articles, we excluded 439 articles because of their publication date (published more than 7 years ago). Subsequently, we read the abstracts of the 338 remaining articles, and, among them, 313 were not included in our final analysis (167 had no relevant information, six were not in English, and 140 matched at least one of the exclusion criteria). Therefore, a total of 25 articles were included in the study. Of these, 11 were literature reviews, one was a prospective cohort study, one was an experimental study, three were case-control studies, three were pre-clinical trials, and six were clinical trials (Figure 1 and Table 1). The articles selected show a relationship between oxidative stress and the possible development of complications in the preterm. Furthermore, given the absence of evidence for the treatment of oxidative stress in the preterm, we have selected three pre-clinical trials that evaluate the use of certain molecules able to prevent the development of neurological, gastroenteric, and respiratory complications (Table 2). In addition, six therapeutic clinical trials were included, reporting evidence of a protective role against the development of oxidative stress and neuronal damage and/or NEC, as the other trials did not comply with the selection criteria (Table 3).

## 4. Discussion

Oxidative stress or, rather, the imbalance between the newborn’s oxidizing and antioxidant factors, seems to play an important role in the onset of the main pathologies of the premature infant, such as BPD, ROP, NEC, IVH, periventricular leukomalacia (PVL), and punctated white matter lesions (PWMLs). Multiple studies have reported that oxidative stress is among the main causes of PWMLs, with which it shares risk factors, such as hyperoxia, hypoxia, ischemia–reperfusion hemorrhage, and maternal/fetal inflammation. Indeed, the microglial inflammatory response in the cerebral white matter can generate free radicals; for this reason, the optimal concentration of oxygen for the resuscitation of preterm infants is strictly monitored [10].

Other studies have shown a close association between oxidative stress and NEC. The first study, which evaluates the global oxidizing/antioxidant status of infants with and without NEC, highlighted how preterms with NEC had higher values of total oxidant status (TOS) and on the oxidative stress index (OSI) than did controls [40,41]. The serum levels of antioxidant capacity (TAC) were measured as described by Erel [42], using the bleaching of the characteristic color of a more stable 2,2′-azino-bis (3-ethylbenz-thiazoline- 6-sulfonic acid) radical cation by antioxidants. Total oxidant status (TOS) was measured as the oxidation of ferrous ion to ferric ion in the presence of various oxidative species in acidic medium and the measurement of the ferric ion by xylenol orange, as described by Erel [43]. The TOS to TAC ratio was defined as the OSI and used as an indicator of the degree of oxidative stress [44]. The second study showed how higher levels of oxidative markers in cord blood plasma were present in preterm infants with NEC [30]. Lavu et al. studied changes in glycogen synthase kinase 3 beta (GSK3β) activity and its regulation by p38 mitogen-actived protein kinase (p38MAPK) in effecting senescence to further delineate the molecular mechanisms involved in senescence. These authors found that OS-induced P-p38MAPK activation is associated with functional downregulation of GSK3β and arrest of cell cycle progression and senescence of amnion cells. Lack of nuclear translocation of β-Cat and its excretion via exosomes further supports the postulation that GSK3β downregulation by p38MAPK might stop cell proliferation preceding cell senescence [25]. This study can be paired with the study by Dutta et al., who found mechanistic differences between PTB and PPROM by revealing differences in fetal membrane redox status, oxidative stress-induced damage, distinct signaling pathways, and senescence activation [35].

Forde et al., underlined how care in a neonatal intensive care unit (NICU) is crucial for preventing the onset of oxidative stress in preterm infants, assessing the impact of kangaroo mother care (KMC; in which a parent holds an infant skin-to-skin on their bare chest for extended periods of time), on physiologic measures of stress (abdominal temperature, heart rate, oxygen saturation, perfusion index, near-infrared spectrometry), oxidative stress, and energy conservation in preterm infants. These authors detected significantly lower levels of urinary allantoin, a biochemical marker of oxidative stress, in preterm infants treated with 1 h of KMC the day prior, compared to controls, who had been treated with incubator care the previous day. This suggests that infants treated with KMC exhibit lower inflammatory tone over time, lending support to the practice of early KMC intervention in the NICU to ultimately reduce stress and promote the health and wellbeing of preterm infants [17].

In a study comparing DNA strand breaks as one of many consequences of DNA oxidation in white blood cells, malondialdehyde (an oxidative stress marker), catalase, superoxide dismutase activity, and total antioxidant capacity (markers of antioxidant defense) in the cord blood plasma of a group of 25 preterms and 25 full-term births, Norishadkam highlighted the greater susceptibility to damage to the DNA of preterms compared to babies born at term [34]. Cai et al., assessed the effect of feeding practices on gut microbiome development and oxidative stress in preterm infants [5]. Preterm infants fed human milk with human milk fortifier or formula-only diets showed a significant increase in F2-isoprostane levels (*p* < 0.05) relative to those fed a diet of human milk with formula. The gut microbiome of the infants fed the human milk with a fortifier diet showed a lower abundance of Veillonella (*p* < 0.05) compared to that of infants fed the human milk with formula diet. The gut microbiome of the infants fed the formula-only diet showed the lowest microbial diversity and the highest relative abundance of *Terrisporobacter* (*p* < 0.05) compared to the gut microbiomes of the other infants. A low abundance of Veillonella might be associated with the increased oxidative stress of infants who were fed breast milk with a fortifier. By contrast, a high abundance of *Terrisporobacter* and *Peptoclostridium* and low bacterial diversity might be associated with increased oxidative stress in infants who are fed formula [15].

These results show that feeding practices affect the bacterial diversity and composition of the gut microbiome, which is associated with oxidative stress in the very low birth weight (VLBW) of preterm infants [8,45]. As reported by Marseglia et al. [21], the 2018 Odzemir study shows how the serum neutrophil-lymphocyte ratio (NLR) appears to have a predictive role in the development of OS-related diseases, as in the case of BPD; the periods of hypoxia and reoxygenation are sensitive, use the activation of neutrophils, and are responsible for the increase in oxidative metabolism, thus forming species reactive to oxygen and nitrogen [ROS and reactive nitrogen species (RNS), respectively] [46]. If these toxic products are not inactivated, their high chemical reactivity leads to damage to a variety of cellular macromolecules, including proteins, carbohydrates, lipids, and nucleic acid; this damage can progress to the death of lung cells [21].

These macromolecules, as described by the National Institutes of Health, are categorized as biomarkers and, as such, are being studied in the context of the evaluation of different biological processes (Biomarkers Definitions Working Group, 2001).

Analytical methods available to study OS include those aimed at detecting a potential risk of oxidative stress, such as non-protein bound iron (NPBI) with its capacity to generate OH through Fenton reaction, and those aimed at detecting direct oxidative damage to lipids, proteins, and DNA (Figure 1). In addition, stress response proteins, ROS-forming enzymes (e.g., xanthine oxidase (XO), uncoupled nitric oxide synthases (NOS), and nicotinamide adenine dinucleotide phosphate (NADPH) oxidase (NOX)), and the activity of antioxidant enzymes (e.g., superoxide dismutase (SOD), catalase, and glutathione peroxidase (GPX)) can be used as OS biomarkers. [47] To better understand the mechanisms underlying oxidative stress, antioxidant defense, and redox signaling, it is essential to assess the protein thiol redox state. However, this state is seldom assessed immunologically because of the inability to distinguish reduced and reversibly oxidized thiols by Western blotting [48,49,50,51,52,53,54].

A modern, underappreciated opportunity exists to use Click PEGylation to realize the transformative power of simple, time and cost-efficient immunological techniques. Click PEGylation harnesses selective, bio-orthogonal Click chemistry to separate reduced and reversibly oxidized thiols by selectively ligating a low molecular weight polyethylene glycol moiety to the redox state of interest [55].

The dangerous effects of FRs are due to their property lack of stability and ability to react with polyunsaturated fatty acids of cell membranes, proteins, polysaccharides, and nucleic acids [56]. Therefore, clinically useful biomarkers should be measured in a reliable and reproducible way (it must be reasonably stable and present in accessible tissue or fluid), but it should also satisfy at least one requirement, such as showing the specificity of the disease, having prognostic power or correlating with disease activity [57]. There are several molecules that could be used, in theory, as biomarkers, but, to date, these are not used in clinical practice. The main commitments are described below.

### 4.1. Molecules that Suffer Damage from FR

#### 4.1.1. Proteins

Several amino acid residues can undergo oxidative modifications, including the oxidation of sulfur-containing amino acid residues, the hydroxylation of aromatic groups, the nitration of tyrosine residues, the chlorination of aromatic groups, or the conversion of some amino acid residues to carbonyl derivatives [29]. Proteins are one of the main targets of ROS and RNS damage. Various amino acid residues can undergo oxidative changes, such as oxidation of sulfur-containing amino acid residues, hydroxylation of aromatic groups, nitration of tyrosine residues, chlorination of aromatic groups, or conversion of some carbonyl-derivative amino acid residues. In the neonatal period, one of the biomarkers typically measured is the oxidation of phenylalanine (Phe). The oxidation of Phe is due to the attack of the hydroxyl radical (OH), which converts Phe into ortho-tyrosine (o-Tyr) or meta-tyrosine (m-Tyr). Moreover, other byproducts of tyrosine, such as 3-nitrotyrosine (3NO2–Tyr) and 3-chlorothyrosine (3Cl–Tyr), are also useful as biomarkers of nitrosative stress and inflammation, respectively [29]. In the presence of peroxynitrite (ONOO), p–Tyr can be converted into 3NO2–Tyr, a specific biomarker for protein nitration [58]. Protein nitration and RNS signaling play a role in cellular functions, such as inflammatory responses and apoptosis [59]. In fact, in another study, the quantification of the 3Cl–Tyr/p–Tyr ratio in human milk samples could be of potential interest for the assessment of inflammation in different clinical scenarios [60]. Similarly, 3Cl–Tyr, formed by the attack of hypochlorous acid on p–Tyr through the action of the enzyme myeloperoxidase (MPO), is considered a useful inflammatory biomarker. The results of these determinations are expressed as ratios, such as the o–Tyr/Phe, m-Tyr/Phe, 3NO2–Tyr/p–Tyr, and 3Cl–Tyr/p–Tyr ratios. These markers have been validated and extensively applied in the clinical setting in various human biofluid matrices, such as amniotic fluid, urine, plasma, cerebrospinal fluid, or human milk in the newborn period [16,24,29,31,36,61,62,63,64]. In urine, in a pre-clinical study conducted on pig pups, we found a significant increase in both urinary o–tyrosine and 8-oxodG levels after 15 min of hyperoxia by resuscitation [62]. Prematurity is associated with protracted oxidative stress, and human milk is partially protective, as there is a reduction in biomarkers in the urine like o–Tyr/Phe [63]

#### 4.1.2. Lipids

The lipid peroxidation process and the damage by its byproducts are among those most described in the literature on free radical damage. This process can take place enzymatically, through the oxidation of arachidonic acid by cyclooxygenase and lipoxygenase, with the formation of prostaglandins, prostacycline, thromboxane, leukotrienes, and lipoxins. All polyunsaturated fatty acids (PUFAs), as such, can undergo this enzymatic oxidation, leading to the formation of protectins, maresins [65], and dihydroxy-PUFAs (diHPUFAs). However, a relevant finding is that we did not identify evidence of the presence of enzymatic oxidation of fatty acid-derived lipid peroxidation markers in preterm infants. Neonatal markers of PUFA peroxidation include isoprostanes (IsoPs) [66,67], isofurans (IsoFs), dihomo-isoprostanes (Dihomo-IsoPs), neuroprostanes (NeuroPs), and neurofurans (NeuroFs). These are non-enzymatic products from the oxidation of amino acid-free radicals and other PUFAs, and are chemically stable. These compounds have been detected electively in lipid-rich tissues, such as brain tissue, which, being rich in polyunsaturated lipids, is highly sensitive to oxidative stress [16,17,18,19,20,21,25,29,30,33,34,35,40,42,43,44,45,46,47,48,49,50,51,52,53,54,55,56,57,58,59,61,65,66,67].

Deshpande and Cai performed a double-blind, randomized controlled trial conducted in very preterm neonates (between 23 and < 28 weeks’ gestation) [68]. This assessed 5 days of parenteral nutrition with a lipid emulsion containing 80% olive oil and 20% soybean oil vs. a pure soybean oil emulsion. The rationale for this study was that olive oil could favor-preterm neonates by reducing oxidative injury, but the authors did not find significant differences. Plasma F2-isoprostane levels were used as a marker of in vivo oxidative stress and lipid peroxidation, and are considered the “gold-standard” biomarker for this parameter [69].

#### 4.1.3. Nucleic Acids

In physiological conditions, DNA is constantly subject to oxidative damage, but DNA repair mechanisms usually correct these alterations. However, oxidative damage to DNA can be complicated by the oxidative alteration of DNA repair enzymes. The oxidation of DNA components by ROS is the main source of damage to DNA, resulting in various modifications, including nucleotide oxidation, filament breaks, base loss, and adduct formation. Hydroxyl radicals can attack the DNA chain and the nitrogen bases of DNA nucleotides or deoxyribose. The main damage for OS to DNA is generally produced by guanosine-based oxidation products, such as 7,8-hydroxy-2′-deoxyguanosine (8-oxodG or 8-OHdG), which can be measured quantitatively in urine, plasma, and cerebrospinal fluid, among others, by high-performance liquid or gas chromatography coupled with mass spectrometry (UPLC–MS/MS) [29,33,58,59,61,65,66,67]. Sánchez-Illana et al., have described a method for the simultaneous detection of a panel of oxidative stress-related biomarkers for the quantification of damage to proteins and DNA in human breast milk samples. The results demonstrate the feasibility of this method for the analysis of a panel of oxidative stress-related biomarkers (2dG, m-Tyr, 3Cl–Tyr), reporting ranges found in human milk [60].

### 4.2. Therapeutical View from Experimental to Clinical Evidence

The choice of which therapeutic treatment as the principal goal for a reduction in the production of free radicals in premature born is still controversial and challenging. In this setting, even if no guidelines are provided, several pre-clinical and clinical trials allow us to identify some substances as potential antioxidants in this population.

#### 4.2.1. Pre-Clinical Trials

Among the pre-clinical studies currently available, the authors of the current manuscripts have identified three main studies. The first, from Romantsik et al., tested the administration of 25 μL of intracerebroventricular α1-microglobulin in rabbit pups. This acts through the binding of the heme group and the reduction in the synthesis of free radicals by its reductase activity. α1-microglobulin is distributed to periventricular cerebellar regions with high plasticity, having a neuroprotective effect against brain damage as a result of IVH preterm [27].

The second pre-clinical study evaluated the antioxidant effect of caffeine related to the state of hyperoxia present in a cohort of rat pups with bronchopulmonary dysplasia. Intravenous caffeine (10 mg/kg) was administered every 48 h, immediately after birth, in order to avoid elevated doses of caffeine outside the therapeutic range that might induce pro-inflammatory responses [70]. This resulted in reduced oxidative damage to DNA, and antagonism of adenosine receptors, a protective action against the response to oxidative stress, and complete interruption of oxidative damage induced by pulmonary hyperoxia. All of the abovementioned features have been found to be linked with a protective role in the development of BPD [26]. Pre-clinical data coming from newborn cavies (rats) has raised the hypothesis of a potential caffeine-related loss of weight. This assumption seems to be underlined by some clinical studies [71,72,73]. However, this is of a temporary nature.

The third pre-clinical study aimed to investigate whether treatment with Ankaferd Blood Stopper^®^ (ABS) reduced the severity of NEC in rat pups in an experimental NEC model. ABS is a folkloric herbal extract used as a hemostatic agent in traditional Turkish medicine [74]. ABS consists of a standardized mixture of *Thymus vulgaris*, *Alpinia officinarum*, *Vitis* *vinifera*, *Glycyrrhiza glabra*, and *Urtica dioicaplant* extracts [75]. ABS influences inflammatory and hemostatic processes via its effect on the endothelium, blood cells, angiogenesis, cellular proliferation, vascular dynamics, and cellular mediators [74,75,76], through its antioxidant, anti-inflammatory, antimicrobial, anti-apoptotic, and wound healing accelerant properties. In this trial, ABS was administered intraperitoneally to the pups in the NEC group. The drug administration protocol started on day 1 of the study at a dose of 2 mL/kg by diluting with 2 mL of saline at a ratio of 1:3. The intervention group resulted in a significantly reduced apoptosis rate and a reduction in the intestinal lesions. Given the above, the antioxidant, anti-inflammatory, and anti-apoptotic intestinal properties reduced the NEC in this specific context [28] [Table 2].

#### 4.2.2. Clinical Trials

Here, we describe the relevant clinical studies, in order of publication. A clinical study conducted by Buonocore at the University of Siena enlisted 1300 newborns with a birth weight of 1500 g or a gestational age of 32 weeks. The investigated cohort was divided into two groups: the experimental group received a daily dose of 100 mg of lactoferrin at standard therapy, while the control group received only standard therapy. Therefore, the main objective was to evaluate the antioxidant effect and the neuroprotective role of lactoferrin and its ability to reduce diseases of free radicals [39]. This is the first study in the literature to address the role of lactoferrin in preterms. It is well established how lactoferrin is an iron-binding glycoprotein with multiple biological functions (iron absorption, anti-inflammatory, immunomodulatory, antioxidant, host defense mechanism, anti-carcinogenicity) [77,78]. A pre-clinical trial showed great promise in the field of neonatology, and for the care of premature newborns, in this model, lactoferrin could protect the preterm rat brain against cerebral hypoxia-ischemia injury [79].

A study still in experimental phase 2, conducted by the University of Siena in 2014, enrolled 100 preterm born neonates with gestational age < 30 weeks, reported that retinoic acid (lutein), a derivative of vitamin A, is a neuroprotective factor. This analysis showed the reduction in the outcome ROP through a dose of 0.28 mg of lutein administered enterally every 6 h, and within the first 36 h of life. These results underline how the inhibition of peroxidation of membrane lipids is of paramount importance for the photoreceptors and neurons [38,80]. Ramani et al., in 2017, suggested that a combination of vitamin A plus retinoic acid might be neuroprotective in extremely preterm mice at higher risk of neurological impairment due to excess oxidative stress [81].

According to a phase 1 study conducted by several US universities in 2015, vitamin E could also be considered a protective element. The purpose of this analysis was to test both the safety and efficacy of administration of one dose of vitamin E, via a tube into the stomach, to extremely preterm infants (less than 27 weeks’ gestation and less than 1000 g weight at birth). This pivotal trial examined whether a single dose of vitamin E would be absorbed into the infants’ bloodstreams, increasing serum α-tocopherol levels in the target range of 1–3 mg/dl. This was found to be linked with a reduction in the incidence of death or neurodevelopmental impairment [37].

Recently, a review summarized and analyzed the state of the art and relation between vitamin A levels and diseases of premature infants. However, although sufficient evidence suggests that vitamin A supplementation is beneficial in preterm infants, the evidence is still lacking for recommended methods for supplementation and dosing of vitamin A. Therefore, in this clinical trial, vitamin A, with its well-known antioxidant effect on lipid membranes, was administered orally at 1500 IU/day for 28 days, and seemed to have a protective effect related to the development of the ROP in relation to the control group, in a study of 254 premature infants weighing less than 1500 g [32].

Melatonin is a naturally occurring neuroendocrine molecule secreted in response to environmental light–dark cycles. Melatonin influences numerous physiological functions, including growth and development, reproduction, and the immune response [82].

Then, acting via specific cell membranes and nuclear receptors, melatonin achieves powerful neuroprotective effect via antioxidant, anti-apoptotic, and anti-inflammatory processes [83,84] and by promoting neuronal and glial development [85,86]. Additional indirect antioxidant effects of melatonin include the up-regulation of antioxidant enzymes and, crucially, the preservation of mitochondrial integrity [87,88].

The University of Pavia has carried out a clinical trial on Melatonin (ME), where researchers investigated the protective role against cerebral ischemia through its powerful antioxidant/inflammatory effects. This study aimed to highlight how ME can prevent brain damage due to premature childbirth. ME was administered orally at a dose of 3 mg/kg/day for 15 days in infants born before gestation of 29 weeks. This was a prospective and randomized study, double-blind controlled, and with two parallel arms. Furthermore, ME was proven to have a good safety profile with no known adverse effects [22].

### 4.3. Future Prospects

The extremely brief half-life of free radicals does not allow for direct measurement of their concentration in clinical settings; therefore, thanks to studies such as those of Stefanovic et al. [7], various analytical strategies have been developed to evaluate the state of oxidation, such as evaluation—both at the extracellular (plasma, serum, urine, amniotic, cerebrospinal fluid) and intracellular (erythrocytes, leukocytes) level—of the oxidoreductive quotient of molecules containing disulfide bridges (SS), such as glutathione in a reduced or oxidized state (GSH or GSSG), or cysteine–cystine (Cys–H or Cys–SS), or the activity of antioxidant enzymes such as SOD, CAT, or GPX. In addition, Stefanovic et al., have developed a new method of evaluation of the redox stage of GSH based on surface-enhanced Raman spectroscopy (SERS), which exploits a silver colloid that improves the GSH signal and allows the accurate measurement of micro-volumes (20 mcl) of blood [7]. This method, associated with the use of UPLC-MS/MS, allows the study of oxidative stress byproducts related to specific tissue components, such as proteins, lipids, carbohydrates, or nucleic acids measured in various body biofluids. Torres-Cuevas et al., have developed a UPLC-MS/MS for recording the profiles of the relative contents of IsoP, IsoF, NeuroP, and NeuroF in a total of 536 urine samples during the first 4 weeks of life in 184 premature infants who did not develop any pathology associated with oxidative stress during the neonatal period [33]. A prospective study of 90 preterm infants in Ain Shams University neonatal intensive care units enrolled infants suffering from food intolerance in the form of abdominal distension, gastric residue, or bloody stool. The first cohort of 45 children was given 20 mg (ME), divided into two daily doses of 10 mg each with a 1-h interval between them, in addition to traditional antibiotic treatment. The second group of 45 children received only standard antibiotic treatment. Both groups were then compared regarding the incidence of necrotizing enterocolitis and the level of the tumor necrosis alpha factor 72 h after starting treatment as a marker of oxidative stress, having a protective role against the NEC [23].

Evidence derived from the literature strongly supports that melatonin is neuroprotective for acute hypoxic-ischemic perinatal brain injury, mediated via its antioxidant, anti-apoptotic, and anti-inflammatory properties. Melatonin is safe in its administration both during pregnancy and after delivery with respect to the newborn, and can reduce white matter brain injury under conditions of chronic fetal hypoxia [89].

Currently, therapeutic strategies to decrease antioxidant cascade, in neonates, are poor and limited. Future prospective studies should be dedicated to verifying the promise of single pharmacotherapy to determine the optimal therapeutic time window for brain protection and, when possible, to decrease the risk of brain damage [Table 3].

The lung microbiome has an important role in the maturation of the immune system [90]; this might act through the production of metabolites, such as tryptophan catabolites, which are agonist of the aryl hydrocarbon receptor (AhR) that regulates the production of antioxidant enzymes [91,92].

Lactobacilli have the ability to metabolize tryptophan into AhR agonists, suggesting an important modulatory role of these bacteria on the OS [93,94]. Moreover, a study in mice reported that the injection of Lactobacilli into the lungs could improve alveolar development [95].

All of these findings indicate a potential role of the microbiome in reducing the OS correlated to BPD. This can be explained by their capacity to reduce macrophage production of pro-inflammatory cytokines, which are responsible for amplifying OS [96].

According to the current literature, the relevance of OS biomarkers in preterm is well established, highlighting the importance of identifying high-risk preterm newborns and predicting their short- and long-term outcomes. Overcoming the technical and economic difficulties that preclude the use of OS biomarkers and the appropriate treatments in clinical practice is challenging, but would lead to an improvement for an accurate evaluation of OS and, consequently, the quality of care of neonatal patients.

## 5. Conclusions

**Figure 2:**
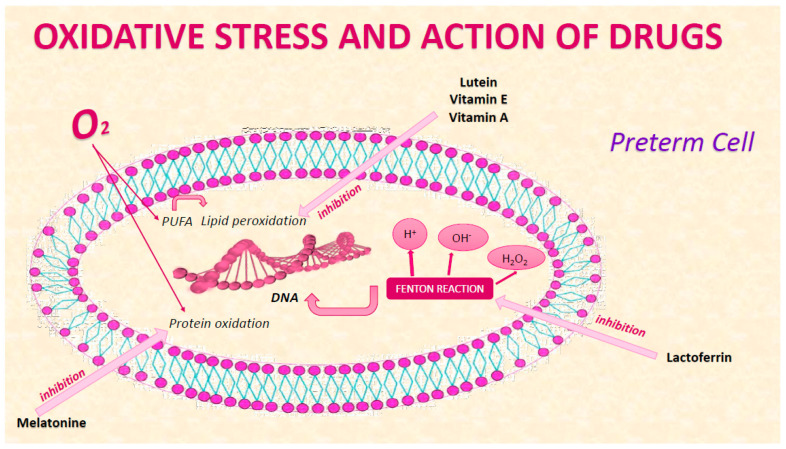
Drug action on cellular oxidative stress of preterm.

The most recent studies show that the relationship between oxidative stress and prematurity is receiving increasing attention from the scientific community. Several biomarkers emerged from our research, such as the products of the peroxidation of polyunsaturated fatty acids (PUFAs), those of the oxidation of phenylalanine, and the hydroxyl radicals that can attack the DNA chain. None of the biomarkers studied and listed above are currently used. Among the most promising drugs, according to our study, are those for the prevention of neurological damage, such as melatonin, retinoid lactoferrin, and vitamin E (Figure 2). The microbiome has an important role in oxidative stress; in fact, the use of lactobacilli might be protective against OS lesions in preterm infants [97]. The search for new biomarkers, the improvement of care within the NICU, and the use of new machines and increasingly precise techniques for the study of oxidative stress products and related pathologies are guiding us towards increasingly targeted interventions (preventive, diagnostic, and therapeutic). Based on this evidence, it is possible to hypothesize that in the coming years, the diagnostic strategies aimed at identifying the risk of the onset of conditions related to oxidative stress will be further refined, and the guidelines for their prevention and treatment will be updated, thereby reducing mortality and morbidity.

## Figures and Tables

**Table 1:** Papers included in the analysis.

Type of Study	Review Article	Experimental Study	Preclinical trial	Clinical trial
2020		DOI: 10.1177/1099800419900231 [17] DOI: 10.1016/j.freeradbiomed.2019.02.032 [5]		ClinicalTrials.gov Identifier: NCT04235673 [22] ClinicalTrials.gov Identifier: NCT04304807 [23]
2019	DOI: 10.1016/j.freeradbiomed.2019.02.019 [6] DOI: 10.1016/j.resp.2019.05.006 [24] DOI: 10.1016/j.freeradbiomed.2019.06.008 [7] DOI: 10.1016/j.freeradbiomed.2019.04.029 [21]	DOI: 10.1093/biolre/ioz119 [25]	DOI: 10.1186/s12931-019-1063-5 [26] DOI: 10.1186/s12974-019-1486-4. [27] DOI: 10.1016/j.biopha.2018.11.023 [28]	
2018	DOI: 10.3389/fped.2018.00369 [10] DOI: 10.3390/antiox7120193 [29] DOI: 10.1155/2018/7397659 [30] DOI: 10.1177/1099800418791028 [16] DOI: 10.1016/j.redox.2018.04.022 [31]			ClinicalTrials.gov Identifier: NCT03154723 [32]
2017	DOI: 10.1016/j.redox.2017.03.011 [33]	DOI: 10.1016/j.mrgentox.2017.10.003 [34]		
2016		DOI: 10.1093/molehr/gav074 [35]		
2015	DOI: 10.1016/j.arcped.2015.05.019 [36]			ClinicalTrials.gov Identifier: NCT01193270 [37]
2014				ClinicalTrials.gov Identifier: NCT02068807 [38]
2013				ClinicalTrials.gov Identifier: NCT01172236 [39]

**Table 2:** Preclinical studies on Substances that prevent oxidative stress in preterm.

Substances under Study	α_1_-Microglobulin	Caffeine	Ankaferd Blood Stopper^®^
Preclinical trials	The heme and A1M confers early protection of the immature brain following preterm intraventricular hemorrhage J Neuroinflammation	Antioxidative effects of caffeine in a hyperoxia-based rat model of bronchopulmonary dysplasia	Therapeutic and preventative effects of ankaferd blood stopper in an experimental necrotizing enterocolitis model
Authors	Olga Romantsik, et al. [27]	Stefanie Endesfelder, et al. [26]	Mehmet Buyuktiryaki, et al. [28]
Year of publication	2019	2019	2019
Cavy (animal)	Rabbits pups	Rats pups	Rats pups
Molecule	Protein	Methylxanthine	Protein
Mechanisms of action	-Heme binding -Reductase activity -Radical scavenging -Binding to mitochondria	-Reduced oxidative DNA damage -Protective interference with the oxidative stress response -Antagonism of adenosine receptors -Full blocking of hyperoxia-induced oxidative	-Significantly reduced apoptosis -Reduction intestinal lesion -Antioxidant, antinflammatory, and antiapoptotic properties
Distribution	Periventricular cerebellar regions with high plasticity (white matter, subventricular zone, corpus callosum, corona radiata, thalamocortical projection)	Pulmonary	Intestinal
Administration	Intracerebroventricular	Intravenous	Intraperitoneal
Posology	25 μL	10 mg/kg every 48 h beginning on the day of birth	2 mL/kg by diluting 2 mL with saline at a ratio of 1:3
Protective role	neuroprotective against brain damage following preterm IVH	BPD	NEC

Radical scavenger α_1_-microglobulin (A1M); bronchopulmonary dysplasia (BPD); necrotizing enterocolitis (NEC).

**Table 3:** Clinical studies on drugs that prevent oxidative stress in preterm.

Drugs	Lactoferrin	Lutein	Vitamin E	Vitamin A	Studies of	Melatonin
Clinical trials	Supplementation with Lactoferrin in Preterm Newborns (lactoprenew)	Evaluation of Antioxidant Activity of Oral Lutein in Preterm and Term Newborn	Vitamin E for Extremely Preterm Infants	Effects of Early Vitamin A Supplementation on the Risk for Retinopathy of Prematurity in Extremely Preterm Infants	Oral Melatonin as Neuroprotectant in Preterm Infants	Effect of Melatonin on Feeding Intolerance and Incidence of Necrotizing Enterocolitis in Preterm Infants
Date	January 2013	February 2014	November 2015	September 2018	January 2020	March 2020
Study phase	4	2	1	Not Applicable	Not Applicable	4
Population	Birthweight ≤ 1500 grand/or gestational age ≤ 32 weeks	Preterm born neonates with gestational age <30 weeks	Preterm infants < 27 weeks’ gestation and < 1000 g birth weight	Premature infants weighing less than 1500 g	Neonates born before 29 + 6-week gestation	Preterm infants < 37 week
Total Population	1300	100	93	262	60	90
Mechanisms of action	Binds iron with high affinity and its structure is unusually resistant to proteolytic degradation. Antioxidant sequestering free ions of Fe^2+^ and thus preventing lipid peroxidation and subsequent milk oxidation	Activity of inhibition of peroxidation of membrane lipids is peculiarly important for the photoreceptors and neurons	Effective lipid membrane antioxidant	Effective lipid membrane antioxidant	Potent antioxidant/-inflammatory effect	potent antioxidant/-inflammatory effect
Posology	100 mg/day	0.28 mg of lutein in two doses: within 6 h	α-tocopheryl acetate 50 IU/kg. 1 dose	1500 IU/day for 28 day	3 mg/kg/day for 15 days	20 mg/day in two doses of 10 mg each with a 1-h interval in between
Administration	Enteral	Enteral	Intragastrical	Enteral	Enteral	Enteral
Protective role	Neuroprotective	Neuroprotective ROP	Reduce the incidence of death or development of neural impairment	ROP	Neuroprotectant for cerebral ischemia	NEC

Retinopathy of prematurity (ROP); necrotizing enterocolitis (NEC).
